# Insights into the role of sialylation in cancer progression and metastasis

**DOI:** 10.1038/s41416-020-01126-7

**Published:** 2020-11-04

**Authors:** Christopher Dobie, Danielle Skropeta

**Affiliations:** 1 School of Chemistry & Molecular Bioscience, Faculty of Science, Medicine & Health; and Molecular Horizons, University of Wollongong, NSW 2522 Wollongong, Australia; 2 Illawarra Health & Medical Research Institute, Wollongong, NSW 2522 Australia

**Keywords:** Glycobiology, Cancer prevention, Metastasis, Tumour biomarkers, Drug discovery and development

## Abstract

Upregulation of sialyltransferases—the enzymes responsible for the addition of sialic acid to growing glycoconjugate chains—and the resultant hypersialylation of up to 40–60% of tumour cell surfaces are established hallmarks of several cancers, including lung, breast, ovarian, pancreatic and prostate cancer. Hypersialylation promotes tumour metastasis by several routes, including enhancing immune evasion and tumour cell survival, and stimulating tumour invasion and migration. The critical role of enzymes that regulate sialic acid in tumour cell growth and metastasis points towards targeting sialylation as a potential new anti-metastatic cancer treatment strategy. Herein, we explore insights into the mechanisms by which hypersialylation plays a role in promoting metastasis, and explore the current state of sialyltransferase inhibitor development.

## Background

The number of new cases of cancer exceeds 18 million globally per year, and is predicted to increase further due to our growing and ageing population; lung cancer is the most common type, followed by breast, prostate and colorectal cancer.^1–3^ The main cause of death in these patients is metastasis, the multistage translocation of a cancer cell to a distant organ where it develops into a new lesion.^4–6^ Although much knowledge has been gained about the metastatic cascade over the past two decades,^7,8^ particularly about the early invasion and migration stages,^9,10^ there is still more to uncover about the genomics of the process, about the circulation and colonisation stages and, in particular, about the role of glycosylation—the attachment of glycans to proteins—in metastasis.^11–13^

Cell-surface glycans have been implicated in tumour aggressiveness, metastasis and chemoresistance,^14,15^ and their presence can be targeted by therapeutic agents, as well as being used for diagnostic purposes; for example, the carbohydrate antigens CA19-9, CA125 and CA15-3 are used for pancreatic, ovarian and breast cancer detection, respectively.^16–19^

Of the various sugars that make up *N*-linked and *O-*linked glycans,^20^ sialic acids at the terminal end of glycans are of critical importance.^21^ In humans, the most common sialic acid is *N*-acetylneuraminic acid (Neu5Ac), which plays an essential role in numerous cellular interactions, including with the extracellular matrix, immune cells, epithelial cells, antibodies and other intercellular processes. The synthesis of sialylated glycans utilises Golgi-resident, membrane-bound sialyltransferase enzymes, of which there are 20 subtypes in humans: all use cytidine monophosphate *N*-acetylneuraminic acid (CMP-Neu5Ac) as the donor.^21^ The sialyltransferases catalyse the formation of a glycosidic linkage between C2 of the sialic acid from the donor and a C3, C6 or C8 hydroxyl of a glycan acceptor, and are named as ST3, ST6 or ST8 subtypes accordingly.^22,23^ These are further categorised based on whether the acceptor sugar is galactose (Gal), *N*-acetylgalactosamine (GalNAc) or another sialic acid (Sia) moiety. The most well-described sialyltransferase in the literature is human ST6Gal I, which produces glycans with the sialic acid linked to C6 of a galactose acceptor.^24^

While 20 sialyltransferase subtypes are a large array, expression of each is regulated across cell types and each presents particular substrate specificities, although with some redundancies.^25^ This has been studied particularly in relation to the most commonly investigated sialyltransferases, ST3Gal I and ST6Gal I.^26^ This review of the human sialyltransferase family by Harduin-Lepers et al. comprehensively details their substrate specificity, sequence alignment, gene expression and roles in *O*-glycan and ganglioside biosynthesis.^25^ On the other side of the sialylation equation, there are four human neuraminidase enzymes (NEU1–4), which cleave sialic acid from glycan chains, thereby also regulating cell-surface sialylation.

Described as a subclass of the glycome, the ‘sialome’ has been linked to a dense forest covering the cell membrane in a varied array of complex sialylated structures^27^ that play vital roles in cell–cell interactions. Dysregulation of this crucial system has far-reaching consequences for cancer, inflammation, infection and immune diseases.^28–30^ A primary example is tumour hypersialylation, an increase in sialic acid residues of up to 40–60% on the surface of cancer cells.^31^ Hypersialylation can occur via the upregulation of sialyltransferases, the downregulation of neuraminidases or a combination of both,^32,33^ and results in an excess of the negatively charged sialic acid on the cell surface. Oncogenes such as Ras and c-Myc have been implicated in the increased transcription of sialyltransferases, among other factors.^32^ The accumulation of sialic acid is linked to immune evasion, along with blocking vital signalling pathways^34^ and reducing the efficacy of chemotherapy and radiotherapy.^35^ Furthermore, hypersialylation promotes tumour metastasis via several routes, including stimulating tumour invasion and migration through integrin-mediated processes,^36,37^ inhibiting Fas-mediated apoptosis^38^ and evading immunosurveillance.^39,40^ Although an increase in sialyltransferase expression promotes a pro-metastatic phenotype in many cancer tissues, it can have the opposite effect on cancers originating in neural tissues, such as glioma, possibly owing to the fact that polysialylation plays a key role in the regulation and regeneration of neural systems.^41^

Several key reviews of sialylation in cancer have been published elsewhere.^32,39,42,43^ In this review, we discuss insights into the specific mechanisms by which altered sialylation promotes cancer metastasis during the stages of invasion, intravasation, circulation, extravasation and colonisation.^44^ Owing to the critical role of sialylation in tumour metastasis, reducing sialylation or regulating sialic acid-mediated processes through small-molecule inhibitors, lectins or blocking antibodies have emerged as potential new cancer treatment strategies. We present the most promising findings, along with future challenges and opportunities for bringing these therapies to the clinic.

## Immune system evasion

**Fig. 1:**
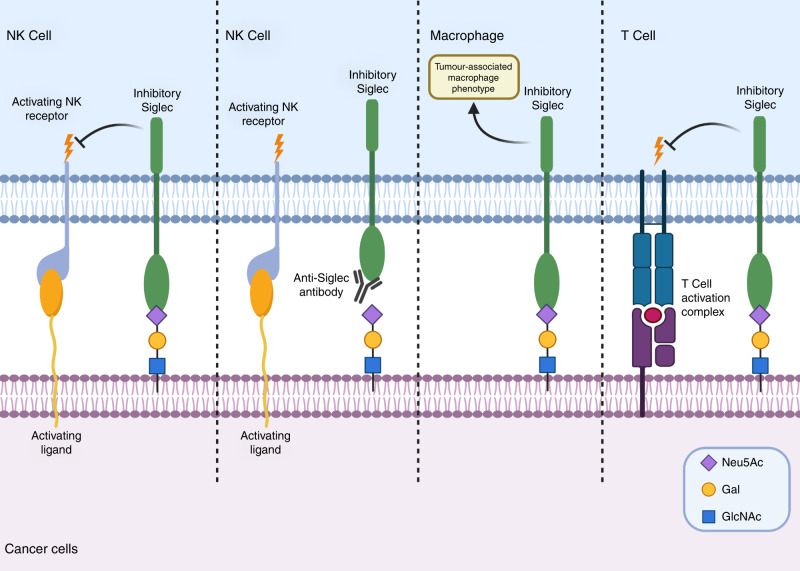
Sialic acid in immune system evasion. Sialoglycans on the hypersialylated cancer cell surface bind to Siglecs on immune cells to mediate immunosuppression, inhibiting the cytotoxicity of natural killer (NK) cells and the activation of T cells, and inducing a tumour-associated macrophage phenotype, to promote continued tumour growth.

For cancer cells to successfully metastasise and spread throughout the body, they must be able to avoid detection and destruction by the immune system. This can be achieved by mimicking the glycosylation patterns of healthy immune cells by employing a ‘self’ signal and thereby avoiding immune attack. Hypersialylation of the surface of cancer cells makes these cells prime ligands for sialic acid-binding immunoglobulin-type lectins (Siglecs), which are found on the surface of immune cells.^45,46^ Once bound to sialylated glycans, Siglecs promote immunosuppressive signalling, thus conferring protection on the tumour cell.^39,47,48^ For example, natural killer (NK)-cell-mediated tumour cell death is inhibited by interactions between NK-expressed Siglec-7 or Siglec-9 and sialylated glycans (Siglec ligands) on tumour cells.^49,50^ Accordingly, monoclonal antibodies targeting Siglec-7 and Siglec-9 have shown promise in both in vitro and in vivo models as they prevent Siglec–Siglec ligand interactions (Fig. 1).^51^ Santegoets et al. showed that glioma cells evade myeloid-derived suppressor cells by expressing ligands for Siglec-3, Siglec-5, Siglec-7 and Siglec-9, the latter two being most abundant.^52^ The binding of Siglec-9-expressing macrophages to a Siglec-9 ligand, such as sialylated mucin-1, has been shown to induce a tumour-associated macrophage phenotype and to ‘educate’ myeloid cells to release factors that promote disease progression (such as interleukin 6 and macrophage colony-stimulating factor),^53^ in studies using soluble mucin-1 and mucin-1 expressed on T47D breast cancer cells.^51,53^ Multiple myeloma cells have also been shown to evade NK cells by binding to Siglec-7 and Siglec-9. However, treatment with neuraminidase to cleave the sialic acid residues or inhibition of sialylation using the sialyltransferase inhibitor 3F_ax_-Neu5Ac^34^ (see later section on ‘Sialyltransferase inhibitors’) enabled primary NK cells to kill multiple myeloma cells.^54,55^

The highly sialylated cell-surface protein CD24, which is overexpressed in some forms of ovarian and breast cancer, binds to Siglec-10 on macrophages, thereby protecting the tumour cells from phagocytotic cell death (Fig. 1).^56^ However, treatment of MCF-7 breast cancer cells with an anti-CD24 monoclonal antibody, or genetic ablation of either CD24 or Siglec-10 restored phagocytosis.^56^ Furthermore, an in vivo murine xenograft model showed a reduction in MCF-7 tumour growth and increased survival in mice that had tumours deficient in CD24.^56^

The specific sialylated ligands for several Siglecs have not been fully characterised. Singh and Choi demonstrated that knockout of the sialyltransferase ST3Gal III in melanoma cells reduced α-2,3-sialylation and the metastatic characteristics of these cells.^57^ Typically, melanoma cells bind to subcapsular sinus macrophages in lymph nodes that express Siglec-1, resulting in colonisation of the nodes.^57^ In a murine colon cancer model established by Shapiro and co-workers, it was noted that α-2,8-linked di-sialic acids bound Siglec-E (a homologue of human Siglec-9) expressed on the surface of macrophages, inhibiting the immune response and allowing increased tumour growth. Overexpression of the sialyltransferase ST8Sia VI in its model decreased the survival time from greater than 6 months to 2–3 months.^58^ Further examples of immune evasion include upregulation of ST6Gal I and consequent α-2,6-sialylation in hepatocarcinoma cells, which was shown to inhibit the proliferation of T cells in the tumour microenvironment, thereby promoting immune evasion (Fig. 1).^59^

## Evading apoptosis and cell death

**Fig. 2:**
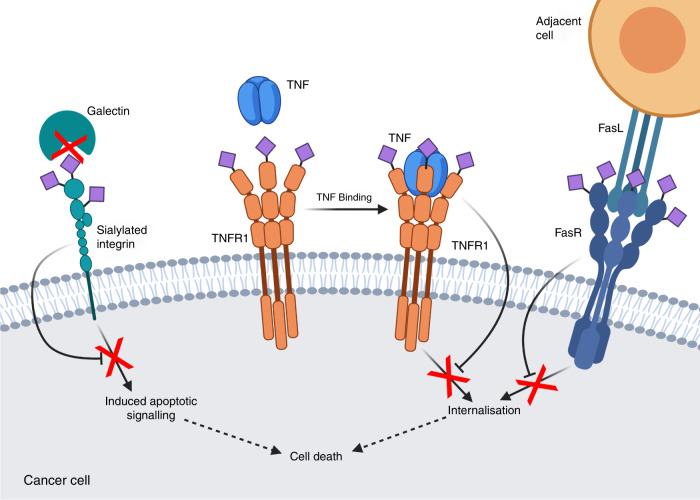
Sialylation in evasion of cell death pathways. Hypersialylation of the Fas receptor (FasR) and tumour necrosis factor (TNF) receptor (TNFR1) inhibits receptor internalisation and downstream cell-death signalling in tumour cells. While galectins are also pro-metastatic, sialylation of extracellular galectin-binding partners such as integrins can block these interactions and reduce galectin-induced apoptotic signalling.

Apoptosis and other mechanisms of cell death are crucial for the body to kill metastatic cells, and so evasion of these processes is critical for proliferation of cancer cells to secondary sites. One of the ways in which cancer cells evade apoptosis and cell death signalling is via ST6Gal-I-mediated hypersialylation of the Fas receptor (FasR). This in turn blocks Fas internalisation and the formation of the death-inducing signalling complex (DISC), thereby disabling apoptotic signalling (Fig. 2).^38^ ST6Gal I upregulation has a similar effect on tumour necrosis factor (TNF)-induced cell death by sialylation of TNF receptor 1, which inhibits its internalisation, thereby preventing the induction of apoptosis and promoting cell survival (Fig. 2).^60^

### Galectins

Galectin-1 is a hypoxia-responsive β-galactoside-binding lectin with elevated expression in many cancers, where it plays a pro-tumorigenic role by promoting angiogenesis and evasion of T-cell- dependent immunity as reviewed elsewhere.^61–65^ Knockout mice models have helped elucidate the role of Gal-1 in promoting immunosuppression and metastasis in breast cancer^66^ and Lewis lung carcinoma.^67^ Inhibition of Gal-1 is being explored as a potential cancer treatment^62^ with calixarene OTX008 reaching Phase 1 clinical trials in 2013, although no further trial data have been reported.^68,69^ Alongside inhibiting galectins, there is an emerging role of sialylation as a negative regulator of galectin binding and function that requires further investigation (Fig. 2).^70–72^ One such example is the sialylation of the fibronectin receptor α5β1-integrin, which impairs its ability to trigger anoikis (apoptosis in response to detachment from the underlying extracellular matrix) by reducing its binding affinity to extracellular Gal-1.^73,74^

Galectin-3 also plays a key role in tumour angiogenesis, migration and invasion,^75^ along with an emerging regulatory role in cancer stemness.^76^ However, Gal-3 has a broad spectrum of activity in tumour growth, where intracellular Gal-3 protects cells from apoptosis, while extracellular Gal-3 can induce apoptosis. Elevated levels of Gal-3 are typically associated with a pro-metastatic tumour phenotype; however, Pereira et al. have reported that reduced Gal-3 expression during breast cancer progression correlated with increased metastasis of 4T1 murine breast cancer cells to the bone marrow.^77^

Sialylation can inhibit the interaction between Gal-3 and its binding partners (integrins, mucins, collagen and fibronectin). Bellis et al. have shown that elevated levels of ST6Gal I in human colon carcinoma result in α-2,6-sialylation of β_1_ integrins that impairs adhesion to extracellular Gal-3 and confers a selective advantage by protecting the tumour against Gal-3-induced apoptosis.^78^ Santos et al. have shown that elevated ST6GalNAc 1 in gastric cancer results in decreased Gal-3 cell-surface- binding sites, leading to intracellular accumulation of Gal-3 and increased chemotherapeutic resistance.^79^ Desialylation of malignant lymphoma cells using an O-glycosylation inhibitor was found by Suzuki et al. to enhance cell adhesion to galectin and inhibited cell invasion.^80^

On the other hand, Murugaesu et al. have shown that ST6GalNAc II acts as a metastasis suppressor in breast cancer by regulating the pro-metastatic role of Gal-3. They found that elevated ST6GalNAc II expression in oestrogen receptor (ER)-negative breast cancers impaired Gal-3 binding to the tumour cell surface, and correlated with reduced lung metastasis and improved survival.^81^ As sialylation was found to regulate Gal-3 binding, it was proposed that galectin inhibitors would be most effective for low ST6GalNAc II-expressing cells. It was found that Gal-3 expression levels did not correlate with clinical outcomes, whereas it was proposed that monitoring of ST6GalNAc II expression could be used to stratify patient treatment or to predict metastasis in ER-negative breast cancers.

Both galectins and sialylation mask the underlying β-galactoside ligand and are characterised as pro-tumorigenic/metastatic, and both are linked to cancer stemness.^76,82^ While they could be expected to work in concert to promote tumour progression, immune evasion and metastasis, the complete picture is far more complex. The intricate relationship between sialylation and galectins extends beyond cancer into cardiovascular and neurodegenerative disease, and highlights a potential role for combination strategies that target both galectins and sialylation in cancer (and other diseases).^65,83^

## Altered adhesion and invasion

**Fig. 3:**
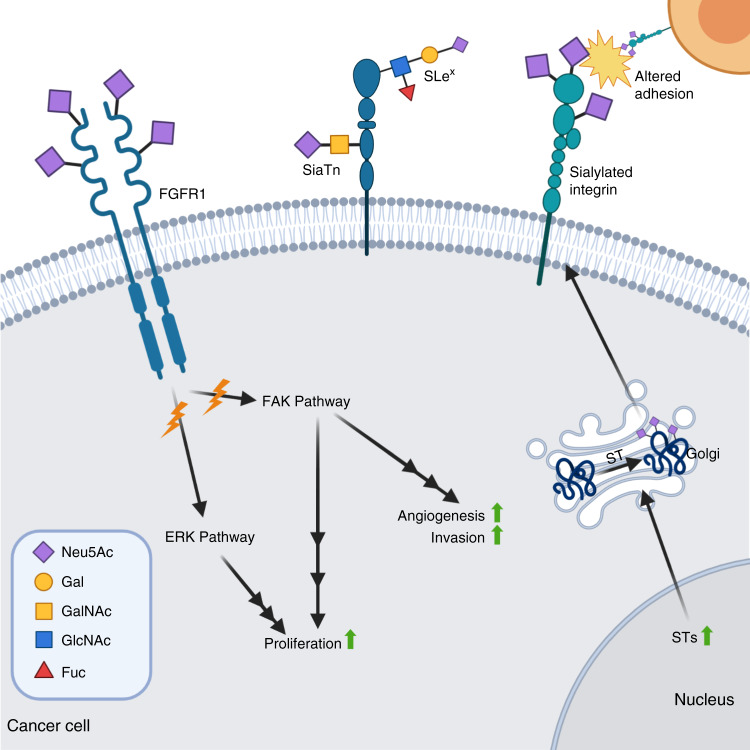
Hypersialylation in altered adhesion and invasion. Hypersialylation of growth-factor receptors such as fibroblast growth-factor receptor (FGFR)1 can activate the receptor, triggering the extracellular signal-regulated kinase (ERK) and focal adhesion kinase (FAK) pathways, leading to increased proliferation, angiogenesis and invasion. Upregulation of multiple sialyltransferases causes hypersialylation of integrins and other adhesion molecules, reducing the stability of tumour masses and thereby increasing the spread of tumour cells.

One key mechanism in metastatic cancer spread is the reduced adhesion between cancer cells and the extracellular matrix (ECM), and other cells in the same tumour mass. This decreases the stability of a tumour mass and allows for cells to more readily separate and invade blood vessels, leading to circulation and potential metastasis at a secondary site. Thus, sialylation of cell–ECM-adhesion molecules, such as integrins, plays a key role in the increased metastatic potential of many cancers through a change to their adhesive properties (Fig. 3).^36^

### Integrins

Glycosylation of integrins, in particular sialylation, controls integrin cell functions. Bellis et al. found that *Ras* upregulates ST6Gal I in colon epithelial cells, leading to increased sialylation of β1-integrin receptors.^84^ Further in vivo studies showed that relative to ST6Gal I non-expressors, ST6Gal I- expressing SW48 colon cancer cells exhibited greater attachment to collagen I and laminin, enhanced migration towards collagen I and increased association with talin.^74^ Similarly, Almarez et al. reported that increased sialylation enhanced integrin-mediated cell mobility on collagen and fibronectin in SW1990 pancreatic cancer cells.^85^ Chiang et al. investigated the effect of a lithocholic acid-based sialyltransferase inhibitor, which decreased sialylation of α5, αv and β1 integrins in A549 lung cancer cells, leading to significantly reduced invasion, as well as suppression of lung metastasis in vivo.^37^

In order to identify the distinct enzymes responsible for sialylation of integrin and other target proteins, Qi et al. developed a series of knockout HeLa cell lines and found that ɑ-2,3-sialylation of β1 integrin occurred in zones of the Golgi apparatus rich in ST3Gal IV and for EGFR in zones containing ST3Gal VI.^86^ The ɑ-2,3-sialylation of N cadherin occurred in Golgi zones containing ST3Gal III, ST3Gal IV and ST3Gal VI, while ST6Gal I was ubiquitously distributed across all zones. This study suggests that distinct α-2,3-sialyltransferases modify specific target proteins, including integrins, and thereby regulate different cellular functions.

β1 Integrin is the main carrier of the sialyl-Tn (STn) epitope, a tumour-associated antigen associated with poor prognosis in several cancers. Sialyl Tn is a truncated *O*-glycan comprising α-2,6-sialylated GalNAc residue on Ser/Thr and synthesised by ST6GalNAc I,^87^ while the sialyl T antigen is a related α-2,3-sialylated disaccharide (Galβ1–3GalNAc) synthesised by ST3Gal I,^88^ and both are highly abundant in carcinoma cell lines.^89^ Fujita et al. used a ST6GalNAc I-expressing MDA-MB-231 cell line and found, contrary to their expectations, that STn expression impaired adhesion of the breast cancer cells to bone marrow stromal cells, fibronectin and type I collagen.^90^ The authors highlighted the limitations of the study and the need to investigate other adenocarcinomas and bone metastasis models. However, Ata and Antonescu^91^ have noted that while sialylation regulates integrin-mediated cell adhesion and migration, this could vary for specific integrin heterodimers and ECM substrate combinations.

### Selectins

**Fig. 4:**
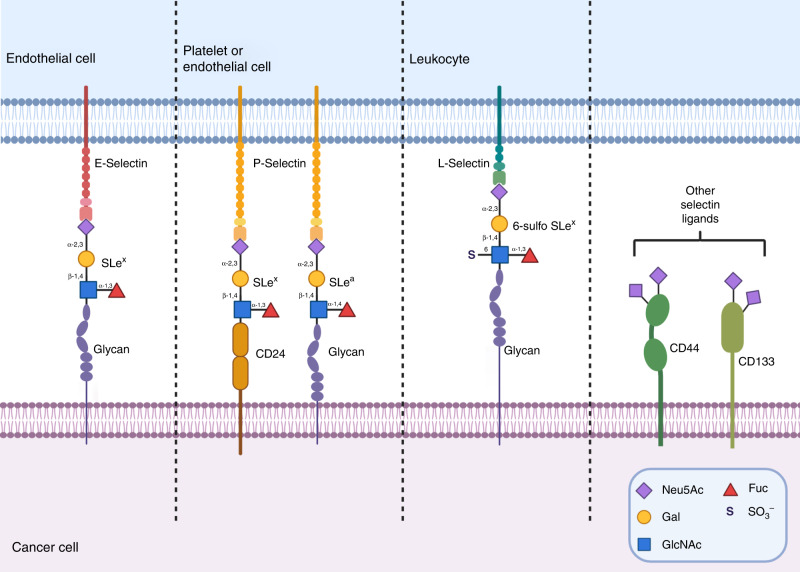
Sialic acids in circulation, extravasation, and colonisation. On the surface of cancer cells, extracellular glycans bearing the sialyl motifs sLe^x^, sLe^a^ and 6-sulfo sLe^x^, and other sialylated glycoproteins such as clusters of differentiation, act as the binding ligands for E-, P- and L-selectin, and play key roles facilitating metastasis to a secondary site.

Selectins are cell-adhesion receptors that bind sialylated glycans and mediate numerous cell- adhesion processes, including leukocyte recruitment, extravasation and homing of lymphocytes, as well as play a key role in cancer metastasis.^92–94^ They are classified as E-, P- and L-selectins based on their expression on endothelial cells, platelets and leukocytes, respectively, with distinct ligand specificities for sialyl Lewis^x^ (sLe^x^) for E-selectin, sLe^a^ or sLe^x^ for P-selectins and the sulfated glycan, sialyl 6-sulfo Le^X^ for L-selectin (Fig. 4).^94–96^ The selectin ligands are found at the terminus of both *N*- and *O*-glycans of mucins, cell-surface antigens such as CD24 and CD44 and the P-selectin glycoprotein ligand-1 (PSGL-1). The increased expression of sialylated selectin ligands on tumours by upregulation of sialyltransferases (and fucosyltransferases) is correlated to enhanced metastasis in many cancers, including melanoma, gastric, pancreatic, colon and lung cancer.^15^ Gomes et al.^97^ found that the expression of ST3Gal IV in MKN45 gastric cancer cells resulted in enhanced synthesis of the sLe^x^ antigen and an increased invasive phenotype both in vitro and in vivo.^97^ In another study, Shen et al.^98^ elevated α-2,3 sialylation by promoting ST3Gal IV expression, which led to increased metastatic potential in gastric cancer cells (particularly MGC-803 cells), as demonstrated by lectin-binding and wound-healing assays. The analysis of tissue samples from 80 patients showed a positive correlation between α-2,3-sialylation and the extent of lymph-node metastasis, cancer stage and depth of tumour invasion.^98^

### Epithelial-to-mesenchymal transition (EMT)

EMT is the process by which polarised, immotile epithelial cells transition into motile mesenchymal cells with a central role in promoting carcinoma invasion and metastasis.^99^ Wu et al. showed that the ST3Gal family of sialyltransferases appears to play an integral role in transforming growth-factor (TGF)-β-associated EMT in ovarian cancer cells.^100^ The study found that treating SKOV-3 and A2780 cells with TGF-β increased the expression of ST3Gal I mRNA, and that this increase led to a decrease in E-cadherin levels and an increase in N-cadherin and vimentin levels; conversely, knockdown of ST3Gal I had the opposite effect, with TGF-β then having no impact whether present or absent. Wen et al. also observed an increase in E cadherin and a corresponding decrease in N cadherin in ES2 ovarian cancer cells when ST3Gal I was knocked down.^101^ By contrast, however, Du et al. found that global inhibition of sialylation promoted EMT in HaCaT keratinocytes, and that sialylation levels decreased upon induction of EMT, but increased again once cells were in the mesenchymal state.^102^ These observations indicate that the process is complex, and that while α-2,3-sialylation can promote EMT, other patterns of sialylation may inhibit certain transitions in the EMT, and hypersialylation becomes a phenotype of cells in the mesenchymal state.

### Growth-factor receptors

In ovarian cancer cells, α-2,6-sialylation of fibroblast growth-factor receptor 1 (FGFR1) by ST6Gal I activates the extracellular signal-regulated protein kinase (ERK) and focal adhesion kinase (FAK) pathways, promoting cell proliferation and migration (Fig. 3).^103^ However, Chandler et al. have observed that *N*-glycosylation and subsequent α-2,6-sialylation of vascular endothelial growth-factor receptor 2 (VEGFR2) at Asn-247 can deactivate this receptor, resulting in the development of abnormal tumour blood vessels; however, further investigation of sialylation at other *N*-glycosylation sites is needed.^104^

In a study of 633 breast cancer patients in China, higher expression of ST6Gal II in cancer tissue relative to healthy tissues was found to be associated with increased tumour stage, decreased survival time and oestrogen receptor (ER)/progesterone receptor (PR)/human epidermal growth-factor receptor 2 (HER2) status.^105^ This observation led the authors to investigate the effects of silencing ST6Gal II in MCF-7 and T47D breast cancer cells, which resulted in the inhibition of cancer progression by arresting cell-cycle progression at G0/G1, as well as inhibiting the expression of the adhesion and migration molecules ICAM-1, VCAM-1, CD24, MMP2, MMP9 and CXCR4. Overall, increased ST6Gal II expression correlated positively with focal adhesion and metastasis pathways, whereas downregulating ST6Gal II expression led to reduced cell adhesion and invasion, leading the authors to propose that ST6Gal II is a potent oncogene and a potential target for treating breast cancer.

Decreased cellular adhesion and increased migration and invasion have been linked to an upregulation of the sialyltransferase ST6GalNAc I, which plays a key role in the synthesis of the sialyl Tn antigen (Fig. 3).^106,107^ Munkley et al.^108^ have highlighted how high levels of STn result in a decrease in the stability of tumour masses, leading to increased metastatic potential. Baeza-Kallee et al.^109^ showed that an increase in the expression of ST8Sia III in glioblastoma cells caused elevated cell proliferation, migration and clonogenicity, whereas treatment with neuraminidase inhibited metastatic potential. In a study of five breast cancer cell lines, one brain cancer cell line and a brain-seeking breast cancer cell line, an increase in total *N*-glycan abundance and overall sialylation levels was associated with increased invasiveness.^110^ The greatest degree of sialylated *N*-glycan expression was observed in the brain-seeking cell line 231BR, highlighting the link between sialylation and cancer metastasis.

In contrast to the examples described above, however, in neural cancers, it has been reported that an increase in the expression of sialyltransferases might have an inhibitory effect on metastasis. For example, ST6GalNAc V is usually expressed in normal brain tissue, and upregulation of this enzyme in other cancers (e.g. breast) can lead to metastasis to the brain.^111^ However, glioma cells show a marked decrease in ST6GalNAc V expression, and transfecting the *ST6GALNAC5* gene into U373MG glioma cells was found to inhibit glioma growth.^112^ Further examples looking at the positive effects of increased sialylation include the intravenous infusion of recombinant ST6Gal I by Lau et al.^113^ They have shown that therapeutic administration of rST6Gal I can regulate de novo inflammatory cell production and dampen the inflammation mediator cascade in a murine model of acute lung inflammation.

## Circulation, extravasation and colonisation

For cancer cells circulating in the bloodstream or lymphatic system to metastasise to a secondary site, they must first tether to cells at the site via the interaction of extracellular molecules. Tethering is followed by ‘rolling’ of the cell against endothelial cells, increasing the number of contacts between the cancer cell and endothelium, resulting in firm adhesion. This allows for extravasation and colonisation at a secondary site to occur.

Circulating tumour cells express several ligands for E-, P- and L-selectins, including sLe^a^, sLe^x^ and CD44, with ST3Gal enzymes most often implicated in their synthesis.^114–117^ Once circulating tumour cells have entered the bloodstream or lymphatic system, these selectin ligands help to tether the cells to the endothelium near the target organ prior to extravasation. CD24 is another ligand for P-selectins expressed on platelets and endothelial cells that is modified with sLe^x^, and aids in P-selectin-dependent rolling of breast and bladder cancers during metastasis to the lungs (Fig. 4).^118^ Sialylation of CD133 (another cell-surface molecule identified as a cancer stem-cell marker)^119^ is also strongly linked to invasion and metastasis.^120^ In 2019, Scott and Drake reported that breast cancers with high levels of sLe^x^ show affinity for E-selectins on endothelial cells, leading to extravasation at potential secondary sites (Fig. 4).^121^

In recent years, E-selectin has emerged as a key regulatory component of the bone marrow haematopoietic stem cell (HSC) vascular niche.^122,123^ Barbier et al. have shown that adhesion to E-selectin in the vascular niche promotes cell survival in acute myeloid leukaemia blasts, while inhibition with a glycomimetic (Uproleselan) reduced this effect and re-sensitised cells to chemotherapy.^124^ SLe^x^ overexpression has also been linked to the progression of pancreatic ductal adenocarcinoma (PDAC),^125^ the spread to massive liver metastasis and significantly worse patient survival.^126,127^ SLe^x^ is expressed on a ligand for P-selectins on the peritoneal mesothelium, which allows for site-specific metastasis of SKOV-3 ovarian cancer cells. P-selectin-bound metastatic cancer stem cells were found to be resistant to shear stress, which highlights the strength of sLe^x^ binding to P-selectins in a circulating fluid environment.^128^ Meanwhile, L-selectins have been implicated in monocyte recruitment, promoting lung metastasis through enhanced extravasation.^93,129^ A study undertaken by Mondal et al.^130^ has demonstrated that ST3Gal IV is the primary sialyltransferase that synthesises sLe^x^ and other selectin ligands on human myeloid leukocytes, facilitating tethering to the vascular endothelium (Fig. 4).^130^

In 2020, Natoni et al. reported that pan-sialyltransferase inhibition in multiple myeloma (MM) cells alters the post-translational sialylation of α4 integrin affecting its affinity for its counter-receptor, as well as reducing interactions between MM cells with E-selectins, MADCAM1 and VCAM1.^131^ Using an in vivo mouse model of aggressive myeloma, sialylation blockade improved survival by enhancing bortezomib sensitivity. The authors propose that sialylation is important for retention of the myeloma cells in the protective bone marrow microenvironment, and that inhibiting sialylation could increase the ratio of circulating to bone marrow-resident MM cells to increase the efficacy of anti-myeloma therapies.

## Altered neuraminidase activity

Whereas hypersialylation of the surface of cancer cells is often attributed to sialyltransferase upregulation, downregulation of neuraminidase (NEU) enzymes, which thereby prevents the cleavage of sialic acids from cell-surface glycoconjugates, also gives rise to increased sialylation. For example, a decrease in NEU activity was identified as a significant contributor to cancer progression by Miyagi et al.,^132^ who also reported in their study on HT-29 colon cancer cells that NEU1 overexpression resulted in a significant reduction in liver metastasis.^132^ The proposed mechanism for reduced metastasis involves a decrease in β4-integrin sialylation, which thereby suppresses cell migration, invasion and adhesion.^133^ Conversely, NEU1 and NEU4 downregulation have been reported to facilitate metastasis in colorectal cancers.^132,134^

Tringali and co-workers have shown that silencing the NEU4 gene in glioblastoma stem cells resulted in the downregulation of ST3Gal III, leading to decreased survival (potentially as a regulatory response to control the overall levels of sialylation), while increased levels of NEU4 increased the survival rate of the cells.^135^ Miyagi and co-workers also demonstrated an increase in serum NEU3 levels amongst cancer patients, as well as the presence of an unidentified NEU3 inhibitor in human serum, which could function to protect cancer cells from sialidase activity.^136^ It could be that the increase in NEU3 is an immune response to reduce cell-surface sialylation, while the NEU3 inhibitor may be expressed by cancers as a defence mechanism.

## Sialylation-based cancer biomarkers

Several biomarkers used in clinical practice take advantage of the hypersialylation of the tumour cell surface, including the widely employed pancreatic cancer marker CA19-9 (sLe^a^) and the sialylated mucin biomarkers CA125 (MUC16) and CA15-3 (MUC1 analogue) used to detect ovarian and breast cancer, respectively.^96,137,138^ Further investigations into additional biomarkers of hypersialylation include the analysis of expression levels of soluble E-selectin and five sialyltransferases (ST3Gal I–IV, ST6Gal I) in 135 surgically treated node-negative breast cancer patients, who found that a high ST3Gal III:ST6Gal I ratio and high levels of soluble E-selectin correlated with a poor prognosis for both relapse-free and overall survival.^139^

In a 2018 study of ovarian cancer using ST6GAL1 mRNA levels from a public database (*n* = 517), along with the analysis of ST6GAL1 protein levels of 204 tumour samples, Wichert et al.^140^ found that high ST6GAL1 mRNA levels correlated with lymphovascular invasion and shorter survival, while high levels of ST6GAL1 protein expression correlated with advanced stage, metastasis and shorter recurrence-free intervals, indicating that ST6GAL1 expression levels could help identify the risk of chemoresistance and metastatic relapse.^140^

MicroRNAs (miRs) are small non-coding RNAs that function as gene regulators, increasingly implicated in the development of drug resistance, along with targeting genes related to cell proliferation, cell cycle and apoptosis.^141^ Liu et al. demonstrated that oncogenic miR-182 and miR-135b mediate the tumorigenesis and invasiveness of colorectal cancer (CRC), as well as resistance to 5-fluorouracil (5-FU),^142^ supported by others.^143^ They also found that miR-182/-135b inversely regulated *ST6GALNAC2* expression via the PI3K/AKT pathway, and suggested that this could be used as a potential predictive marker for CRC.^144^ However, the picture around ST6GalNAc II expression in cancer is far from clear. The *ST6GALNAC2* gene is downregulated in ER-negative breast cancer tumours as described earlier,^81^ while mutations in the *ST6GALNAC2* gene (along with B3GNT2 and B4GALT2) have been observed in patient-derived CRC cells.^145^ Yet, in a 2020 glycoproteomic study, the *ST6GALNAC2* gene was highly upregulated across all oncogenic cell lines investigated, compared with other glycosylation proteins (including other sialyltransferases) that were downregulated in KRAS and HER2 clusters, but upregulated in BRAF, AKT, EGFR and MEK oncogenic clusters.^146^ While there may be potential for ST6GalNAc II as a cancer biomarker, further studies are needed.

Patents on sialylation-based biomarkers are largely based on either detection of the sialyltransferase enzyme (or related gene) or sialylated products such as the tumour-associated sialyl Tn antigen discussed earlier (Fig. 3). ST3Gal I has been patented as a biomarker for predicting prognosis and/or monitoring progression of prostate cancer.^147^ ST3Gal I is also one of the genes identified in a 2014 patent that is expressed in circulating tumour cells and used to differentiate these cells from primary tumour cells, as well as being highlighted as a potential target to inhibit metastasis.^148^

## Sialyltransferase inhibitors

### Small-molecule inhibitors

**Fig. 5:**
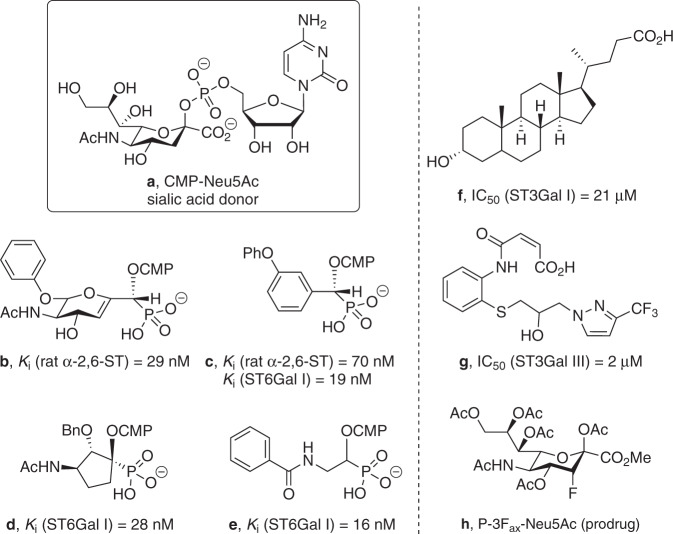
Structures of leading sialyltransferase inhibitors from design (transition-state analogues), natural products and high-throughput screening (compounds). **a** The natural sialic acid donor CMP-Neu5Ac; **b** early transition-state analogue from Schmidt et al.^150^; **c** aryl derivative from Skropeta et al.^153^; **d** cyclopentyl analogue from Li et al.^154^; **e** amide-linked analogue from Guo et al.^156^; **f** natural lithocholic acid; **g** pyrazole compound identified by Rillahan et al.^172^; and **h** the global metabolic ST inhibitor, peracetylated 3F_ax_-Neu5Ac.

The earliest methods for controlling sialylation used small-molecule inhibitors of sialyltransferases. The most well-known class of sialyltransferase inhibitors includes those that mimic the transition state of the sialyl transfer step of the enzyme-catalysed mechanism based on the activated form of the sialic acid donor CMP-Neu5Ac (Fig. 5a).^149^ This class of sialyltransferase inhibitor was pioneered by Schmidt,^150,151^ with 2,3-didehydro-2-deoxyneuraminic acid (DANA, Neu5Ac2en)^152^ and aryl-based phosphodiester compounds shown in Fig. 5b (*K*_i_ = 29 nM, rat α-2,6-ST) and Fig. 5c (*K*_i_ = 70 nM, rat α-2,6-ST; *K*_i_ = 19 nM, hST6Gal I)^153,154^ that were developed two decades ago, still amongst the most potent agents used today.^149^ Numerous analogues of these CMP-based sialyltransferase inhibitors have been explored over the past 5–10 years with a range of activities,^155^ including the cyclopentyl derivative (Fig. 5d; *K*_i_ = 28 nM, hST6Gal I)^154^ and benzamide derivative (Fig. 5e; *K*_i_ = 16 nM, hST6Gal I),^156^ as well as high-affinity fluorescently labelled analogues of the 2,3-didehydro-2-deoxyneuraminic acid compounds shown in Fig. 5b.^157^

Although they show excellent potency, there are potential pharmacokinetic issues with these highly polar phosphodiester-based inhibitors in terms of cell permeability, stability and potential susceptibility to phosphatase cleavage in vivo.^158^ To address these issues, several groups have sought to replace the charged phosphodiester linker with a neutral bioisostere, such as an amide, sulfonamide, carbamate or 1,2,3-triazole to improve both drug likeness and synthetic accessibility.^158–162^ The publication of the crystal structures of porcine ST3Gal I^163^ and human ST6Gal I,^164^ ST8Sia III^165^ and ST6GalNAc I^166^ has enabled the use of structure-based design to improve selectivity without compromising potency. A new series of carbamate-linked ST inhibitors have been recently described.^167^

### Screening for natural and synthetic sialyltransferase inhibitors

A number of natural products with sialyltransferase inhibitory activity in the low micromolar range have been identified, including the soybean-derived soyasaponin I, which is active against ST3Gal I, ginsenosides, which inhibit both α-2,3- and α-2,6-sialylation in HepG2 liver cancer cells^168^ and lithocholic acid, which also inhibits ST3Gal I (Fig. 5f: IC_50_ = 21 µM, ST3Gal I).^169^ Further cell-permeable derivatives of lithocholic acid have been developed, the most promising of which (Lith-O-Asp) suppresses invasion and metastasis of lung cancer cells by inhibiting FAK/paxillin and integrin-mediated signalling (patented in 2016).^37,169–171^ Along with screening natural products, high-throughput screening (HTS) has also been explored. Rillahan et al.^172^ developed a HTS platform involving a fluorescence polarisation assay to screen for both sialyltransferase and fucosyltransferase inhibitors (ST6Gal I, ST3Gal I, ST3Gal III, FUT6 and FUT7). The lead sialyltransferase inhibitor from their screen was pyrazole (Fig. 5g, referred to as HAN00305), which demonstrated high selectivity towards ST3Gal III (*K*_i_ = 1.7 μM).^172^

### Peracetylated 3F_ax_-Neu5Ac

Glycocalyx engineering pioneered by Reutter^173,174^ and Bertozzi^175–178^ exploits the promiscuity of glycosyltransferases by feeding artificial sugar biosynthesis precursors into a cell, resulting in the presentation of modified sialic acids on the cell surface that can be used for a variety of purposes. This technique has the potential to revolutionise cancer diagnostics and treatments, and has been widely reviewed elsewhere.^179–181^ Extending this concept, in 2012, Paulson et al. developed the cell-permeable peracetylated 3F_ax_-Neu5Ac (Fig. 5h), which acts as a global metabolic inhibitor of sialylation.^54,182^ The fluorinated prodrug is converted intracellularly into the active inhibitor CMP-3F_ax_-Neu5Ac using the cell’s own biosynthetic machinery, and globally inhibits all sialyltransferase enzymes, while concurrently interfering with sialic acid biosynthesis inside the cell, thereby reducing overall sialylation. However, when the peracetylated 3F_ax_-Neu5Ac (Fig. 5h) was tested in vivo in a murine model, the global inhibition of sialylation caused kidney and liver dysfunction, highlighting the need for selective inhibitors of sialyltransferase enzymes that target the key subtypes upregulated in cancer.^183^

The delivery of peracetylated 3F_ax_-Neu5Ac (Fig. 5h) encapsulated into tumour-targeting nanoparticles has been shown by Bull et al. to prevent metastasis in a murine lung cancer model while bypassing the toxicity issues of global inhibition of sialylation.^184^ Bull et al. have also explored intra-tumoural injection of 3F_ax_-Neu5Ac that was shown to suppress tumour growth by promoting T-cell-mediated immunity.^185^ Further derivatives of 3F_ax_-Neu5Ac have been patented for a range of potential applications in cancer.^186–188^ At the same time, peracetylated 3F_ax_-Neu5Ac, along with several natural product sialyltransferase inhibitors (e.g. soyasaponin and lithocholic acid), is commercially available, providing valuable tools in the further development of new and improved sialyltransferase inhibitors.

## Targeting sialic-based interactions of siglecs, selectins and galectins

A review by Cagnoni et al.^64^ outlined therapeutic strategies to disrupt Siglec–glycan interactions to control metastasis and inflammation. These approaches include a highly sulfated Neu5Ac derivative NMSO3 that inhibited P-selectin-mediated tumour cell adhesion in human promyelocytic leukaemia,^189^ as well as C-9-modified Neu5Ac derivatives with enhanced affinity for human CD22.^190,191^ The latter example was further modified at the C-2 position by attaching a doxorubicin-loaded liposome, which resulted in a significant increase in survival rates in a xenograft model of human B-cell lymphoma.^192^

Cagnoni’s review also highlighted strategies targeting galectin–glycan interactions that have been explored, including synthetic lactose and lactosamine-based derivatives with improved bioavailability over the natural ligand, which is sensitive to hydrolysis.^64^ Lead compounds such as thiodigalactoside (TDG) have been shown to reduce tumour progression and metastasis in murine models of breast and colon cancer via Gal-1 inhibition.^193,194^ TDG is widely used as a tool in galectin studies to block sugar-binding activity.^195^

Novel routes for targeting Siglec–sialic-acid interactions were reviewed by Daly et al. in 2019,^50^ including Bertozzi’s approach to target and de-sialylate cancer cells using a sialidase (from *Vibrio cholera*) conjugated to the HER2-targeting antibody Trastuzumab.^196^ The antibody–sialidase conjugate exhibited enhanced tumour cell killing in HER2-positive breast cancer cells compared with the antibody alone. Further examples of sialic acid analogues as probes of sialidases and Siglecs were reported in 2020 by Liu et al., including sialic acid clusters with high affinity for Siglecs, as well as fluorophore-labelled sialic acid analogues for imaging cell-surface sialylation.^197^

Adhesion inhibitors that block the interaction between selectins and sialylated ligands expressed on the tumour cell surface are also being actively explored.^198^ A variety of approaches have been investigated, including sulfated oligosaccharides such as heparin and dermatan sulfate, as well as sLe^x^-based glycomimetics and glycopeptides. Uproleselan (GMI-1271) is a small-molecule glycomimetic and E-selectin-specific antagonist that inhibits binding of cells to E-selectin. Uproleselan entered Phase 3 clinical trials in 2019 (NCT03616470) in combination with chemotherapy to treat 380 patients with relapsed/refractory acute myeloid leukaemia (AML).^199^ Another compound from the same company (GMI-1359) has been developed as a dual inhibitor of both E-selectin and CXCR4, and is being investigated as an adjuvant to taxane-based therapy to reduce bone metastases in men with castration-resistant prostate cancer.^200^

Following a different approach, Racotumomab (trade name Vaxira) is a therapeutic vaccine that triggers an immune response against the tumour antigen *N*-glycolyl GM3 ganglioside (NeuGcGM3), which completed Phase 2 clinical trials against small-cell lung cancer in 2014 (NCT01240447) with further trials ongoing for neuroblastoma (NCT02998983).^201,202^ NeuGc is an immunogenic, non-human sialic acid that is abundant in almost all other mammals including non-human primates, as well as highly expressed on the surface of aggressive cancers.^203,204^ Humans cannot synthesise NeuGc; therefore, its incorporation into tumour cells is from dietary sources, in particular red meat.^205,206^ Varki and co-workers have led the field in understanding NeuGc biology and its role in disease,^207^ paving the way for others to explore NeuGc-based approaches in immunotherapy and as a cancer biomarker.^208^

## Glycomic profiling of cell-surface sialylation

The development of mass spectrometry-based *N*-glycan profiling over the last decade has enabled the simultaneous analysis of hundreds of cell-surface glycans revealing rich information regarding disease-based changes in cell-surface glycosylation.^209^ In order to facilitate high-throughput analysis, the terminal sialic acids are sometimes removed (via neuraminidase treatment) prior to glycan analysis to simplify the overall analysis procedure. While this improves efficiency, complementary methods can be used to provide in-depth analysis of intact sialylated species to reveal the dynamic process of cell-surface sialylation throughout the various stages of cancer aggressiveness.^210^ Cell-surface-capture methods such as those developed by Wollscheid et al. are used to covalently label hundreds of *N*-glycans on live cells to generate a global view of the glycoproteome.^211,212^ Activated ion electron-transfer dissociation (AI-ETD) is a mass-spectrometry- based glycopeptide-profiling method that enables large-scale analysis of intact glycopeptides including sialylated glycans.^213,214^

A 2012 glycoproteomic study from Yarema’s group on SW1990 pancreatic cancer cells revealed that bulk metabolic flux through the sialic acid pathway markedly increased sialylation of certain proteins more than twofold.^85^ The increased sialylation enhanced CD44-mediated adhesion to selectins and integrin-mediated cell mobility, indicating that cells can promote metastasis by controlling protein sialylation via metabolic flux. Using glycoproteomic analysis, Packer, Hancock and Fanayan have identified *N*-glycosylation differences in colorectal cancer that correlate with a more metastatic and aggressive phenotype.^215^ They compared the *N-*glycan profiles of membrane proteins from metastatic LIM1215, moderately differentiated LIM1899 and poorly differentiated LIM2405 colorectal cancer cell lines, and found a dominance of high mannose-type glycan structures (70–90%) and high relative abundance of α-2,6-linked sialic acid containing *N*-glycans in all three cell lines. On the other hand, α-2,3-sialylation was only observed in the LIM2405 cell line, indicating that glycosylation profiles may be able to differentiate between disease stages in colorectal cancer in the future.

Along with sialylation, fucosylation also plays a key role in assembly of the sLe^x^ ligand required for E-selectin binding to a cell. In particular, Fut3/Fut6 are linked to promotion of bone metastasis. Using *N*-glycoprotein-capture mass spectrometry, Esposito et al. identified CD44 as the top candidate substrate of human Fut enzymes in MDA-MB-231 and SUM159-M1a breast cancer cells.^216^ However, a CRISPR/Cas9 CD44 knockout in BM2 cells did not decrease E-selectin binding in vitro or inhibit bone metastasis in vivo, implying that murine models of bone metastasis may not be E-selectin dependent.

Using a combination of glycomic analysis with lectin-staining and cell studies, Amano et al.^73^ have shown that tumour suppressor p16(INK4a) modulates cell-surface sialylation and galectin expression to induce anoikis in human Capan-1 pancreatic carcinoma cells by downregulation of key enzymes involved in sialic acid biosynthesis, in particular the bifunctional UDP-N-acetylglucosamine 2-epimerase/N-acetylmannosamine kinase (GNE). This provides an additional means of controlling cell-surface α-2,6-sialylation via regulation of sialic acid biosynthesis rather than ST6GAL1 expression.

## Conclusions

The ability of tumours to mimic host-like cell-surface sialylation—in particular, hypersialylation—is a key element of cancer progression and aggressiveness by enabling evasion of the immunosurveillance system and cell death pathways, while increasing the capacity for metastasis. Hypersialylation of cancer cells also plays a cytoprotective role and contributes to chemotherapy and radiotherapy resistance in several cancers via mechanisms that are still being explored, but believed to be partly due to the added physical barrier of excess sialic acid, both absorbing ionising radiation and blocking diffusion of drug molecules into the cell. It has been shown that increased sialylation of cancer cells can confer resistance to chemotherapeutics such as paclitaxel and cisplatin.^217–220^ To date, no specific hypersialylation mechanisms that promote radiotherapy resistance have been identified, although correlations have been observed, which implicate the importance of STs in radioresistance, especially in colorectal cancers.^221–224^ Further investigation into these sialic acid-related processes is needed to enable the design of effective anti-metastatic agents that target the key sialyltransferase subtypes involved, with further potential to disarm resistance mechanisms.

Further research into translating knowledge of tumoural cell-surface glycosylation into diagnostic applications is ongoing and described as ‘the second golden age for glycomics’.^19,225–227^ Powerful new multiplexed glycoproteomic approaches are emerging involving large-scale analysis of intact sialylated glycans in a linkage-specific manner that are rapidly expanding our understanding of the cell-surface glycoproteome.^209,228^ There are also exciting prospects to use metabolic glycoengineering to exploit cancer cell metabolism for theranostic applications.^229,230^ Although the opportunities for developing new sialylation-based cancer biomarkers and therapeutics look promising, many challenges remain to be addressed. These challenges include the non-specific nature of protein sialylation,^209,231^ the roles of extracellular sialyltransferases in remodelling cell-surface glycans^232,233^ and a greater understanding of potential compounding genetic and environmental effects on sialylation levels,^234,235^ including diet,^236,237^ exercise,^238^ ageing,^239^ alcoholism^240^ and stress.^241,242^ Future genome-wide association studies (GWAS) can also help reveal whether sialyltransferase genes are statistically associated with predisposition to cancer, metastasis and radio-/chemotherapeutic resistance.

Currently, ST3Gal I and ST6Gal I are the most widely studied sialyltransferases and are consequently also the most commonly investigated and targeted of these enzymes in cancer and metastasis. Much opportunity, therefore, remains in terms of wider studies on the entire panel of 20 human sialyltransferases and their different (or overlapping) roles in cancer progression. On this note, it is essential for sialyltransferase inhibitors to be subtype-selective if they are to proceed to clinical trials, due to potential off-target effects on the liver and kidneys, such as those exhibited by the pan-inhibitor peracetylated 3-F_ax_-Neu5Ac. Along with selectivity, cell permeability and bioavailability are also important elements to address regarding the development of clinically relevant selective sialyltransferase inhibitors as potential cancer treatments. There is much to be gained from the ability to regulate sialylation in disease and, in particular, in cancer metastasis, but also still much to explore to deepen our understanding of sialylation in cancer progression and metastasis.

## Data Availability

Not applicable.
